# Nonislet Cell Tumor Hypoglycemia

**DOI:** 10.1155/2013/308086

**Published:** 2013-10-01

**Authors:** Johnson Thomas, Salini C. Kumar

**Affiliations:** Department of Endocrinology, Diabetes & Metabolism, Nassau University Medical Center, East Meadow, NY 11554, USA

## Abstract

Nonislet cell tumor hypoglycemia (NICTH) is a rare cause of hypoglycemia. It is characterized by increased glucose utilization by tissues mediated by a tumor resulting in hypoglycemia. NICTH is usually seen in large mesenchymal tumors including tumors involving the GI tract. Here we will discuss a case, its pathophysiology, and recent advances in the management of NICTH. Our patient was diagnosed with poorly differentiated squamous cell carcinoma of esophagus. He continued to be hypoglycemic even after starting continuous tube feeds and D5W. General workup for hypoglycemia was negative and insulin-like growth factor II (IGF II) was in the normal range. Hypoglycemia secondary to “big” IGF II was considered, and patient was started on steroids. His hypoglycemia resolved within a day of treatment with steroids. Initially patient had hypoglycemia unawareness, which he regained after maintaining euglycemia for 48 hours.

## 1. Introduction

NICTH was first described by Nadler and Wolfer in a patient with hepatocellular carcinoma as early as 1929 [1]. In 1988, Daughaday et al. demonstrated that tumor-induced hypoglycemia was associated with abnormal pro-IGF II (“big” IGF II) acting via the insulin receptor [2]. Being a rare disease, the true incidence of NICTH is not known. But it is estimated to be one per million people years [3]. Most common cancers causing NICTH are tumors of the GI tract, lungs, pancreas, adrenal, and ovary.  Table 1 [3–5] shows the list of tumors associated with NICTH.

## 2. Case Report

A 63-year-old Caucasian male with poorly differentiated squamous cell carcinoma of esophagus diagnosed 45 days ago with metastasis to lung and liver came to emergency room with dizziness. His fingerstick glucose was 27 mg/dL in the emergency room with corresponding plasma glucose of 19 mg/dL despite PEG tube feeding with Pivot 1.5 at 20 mL/hr. Patient was started on D5W at 100 mL/hr and admitted to medicine floor. He denied previous history of diabetes or use of oral hypoglycemic agents and insulin. He was not on steroids prior to admission.

On physical examination, vitals were within normal limits. Positive findings included right upper quadrant mass and PEG tube. No leak from PEG tube was appreciated.

Patient continued to be hypoglycemic while on D5W and Pivot. During these hypoglycemic episodes, patient denied dizziness, diaphoresis, palpitation, chest pain, tremor, or weakness. Patient was also conscious and fully awake even when his blood glucose was below 30 mg/dL. Patient's feed was changed to Jevity (1.5 Cal/mL) at 80 mL/hr and D70 at 17 mL/hr through PEG tube. This regimen provided 3,851 calories per day. Still patient continued to be hypoglycemic.

Significant labs include potassium of 3.1 mEq/L and bilirubin of 1.4 mg/dL. Workup for hypoglycemia is included in Table 2.

**Figure 1:**
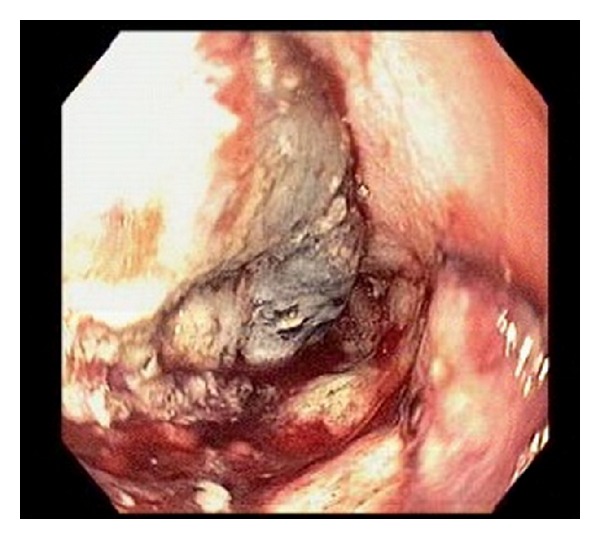
EGD: fungating mass at gastroesophageal junction.

CT scan of thorax and abdomen showed irregular eccentric circumferential thickening of the lower esophagus, multiple bilobar hepatic metastases, and subcentimeter nodule at each lung base. EGD done prior to admission showed a 6 cm fungating mass at gastroesophageal junction (Figure 1). 

Hypoglycemia secondary to “big” IGF II was suspected, and patient was started on prednisone 40 mg once a day. 24 hours after starting prednisone, he became euglycemic, and we were able to taper the tube feeds. Due to poor prognosis, he was discharged to hospice on prednisone. 

## 3. Discussion

NICTH is a rare cause of hypoglycemia. There are multiple mechanisms causing NICTH. Possible pathophysiology includes increased secretion of “big” IGF II which acts on insulin receptor, tumor invasion of liver, and adrenal glands blocking counterregulatory mechanisms to hypoglycemia, tumor producing insulin, and increased glucose utilization by the tumor and antibodies against insulin or insulin receptor.

### 3.1. IGF II Induced Hypoglycemia

There are two isoforms of insulin receptor—isoform A and isoform B. They are created by differential splicing of exon 11 of insulin receptor gene. Isoform A is expressed more in fetal tissues and in malignancies. Isoform B is more seen in tissues which are important in glucose metabolism like liver, fat, and muscles [6].

**Figure 2:**
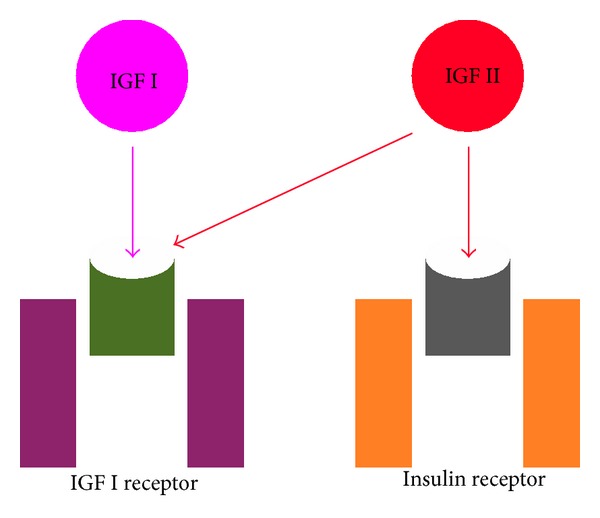
Mechanism of action of IGF II.

IGFs I and II are structurally related to insulin. Their actions are mediated through IGF I receptor. IGF II can also interact directly with insulin receptor (Figure 2). Both IGF II and “big” IGF II are capable of inducing phosphorylation of partially purified preparations of insulin receptor [7]. “Big” IGF II has higher affinity for insulin receptor and lower affinity to its binding proteins [8]. This can lead to actions similar to insulin. Potency for lowering glucose is 10 times lower for IGFs when compared to insulin. In normal subjects concentration of IGFs in the serum is about 100 times that of insulin. Almost all of it, that is, 99%, is bound [7]. As with any hormone, the physiological activity is mediated by the free hormone. Hence IGFs under physiological circumstances do not cause clinically significant hypoglycemia.

 Initiating event in NICTH is the overexpression of pre-pro IGF II gene by the tumor. In a normal individual, the translated peptide undergoes glycosylation. Posttranslational modifications may be impaired in tumors because of insufficient quantity of enzymes required for this process resulting in “big” IGF II [4]. Tumor-derived “big” IGF II primarily forms smaller binary complexes with IGF binding protein, and a greater fraction stays in the free unbound form. Unlike the ternary form, these binary forms can cross the capillary membrane and can act on insulin receptors. This results in inhibition of glycogenolysis, gluconeogenesis, decreased lipolysis, and increased peripheral glucose utilization.

### 3.2. Diagnosis

IGF II in a patient with NICTH will be mostly in the normal range, but it could be above or below the normal range also [9]. Thus, measuring total IGF II will not provide a meaningful result. In a study by Hizuka et al. [5], out of 44 patients with NICTH 31 had “big IGF II” and only 13 patients had elevated IGF II. 

Measuring “big” IGF II is difficult as there is no readily available test. The gold standard for detection of “big” IGF II is size exclusion chromatography. Antibodies against E region of proinsulin-like growth factor II can also be used to detect abnormal IGF II [9]. IGF II/IGF I ratio is also elevated in NICTH secondary to “big” IGF II [5].

Other laboratory results that support the diagnosis include a low insulin and c-peptide level during hypoglycemia, low beta hydroxybutyrate, and hypokalemia. 

### 3.3. Treatment

As with any episode of hypoglycemia, immediate aim is to achieve euglycemia. Depending on the severity of hypoglycemia, patient can be given glucose tablets, fruit juice, dextrose infusion, or glucagon. Definitive treatment depends on the nature of the tumor. If the tumor is resectable, surgery is often curative. Otherwise, chemotherapy, embolization, or radiation can be considered with variable success rate in controlling hypoglycemia. 

Medical management of NICTH includes diazoxide, octreotide, glucagon infusion, steroids, and recombinant growth hormone. Diazoxide works by activating potassium channels thereby decreasing insulin secretion. It can be given in divided doses up to 1200 mg/day. Diazoxide is particularly useful in tumors secreting insulin. If NICTH is due to “big” IGF II, success is limited as insulin level is already low in this condition. Octreotide is a somatostatin analogue which inhibits the release of growth hormone, insulin, gastrin, VIP, glucagon, secretin, motilin, and pancreatic polypeptide. It can be given subcutaneous injections 150–250 mcg 3 times/day. Octreotide is more successful in controlling symptoms of VIPomas, carcinoids, and glucoganomas when compared to NICTH [10]. Steroids are the mainstay of treatment for NICTH. Steroids decrease the amount of “big” IGF II by converting pre-pro IGF into IGF II. They also reestablish the normal IGF to IGFBP ratio [11, 12]. Recombinant growth hormone at supraphysiological doses increases IGFBP and ALS, thereby increasing ternary complex and preventing interaction with insulin receptor ultimately decreasing chances of hypoglycemia [11–13]. But it also increases total IGF which can be hazardous in the long term. Many tumors have IGF receptors, and they may grow in response to this elevated IGF level.

## 4. Conclusion

NICTH is a rare cause of hypoglycemia. It should be suspected in a patient with hypoglycemia and a tumor without any obvious cause. Steroids with or without recombinant growth hormone can prevent recurrent hypoglycemia. A multidisciplinary team approach is required in the optimal management. If the tumor is not resectable and poor prognosis is confirmed, hospice should be considered.

## Figures and Tables

**Table 1:** Tumors associated with NICTH.

GI tract tumors: esophagus, stomach, pancreas, liver, and colon	
Endocrine tumors: adrenal cortical cancer, pheochromocytoma, thyroid, and carcinoid	
Reproductive tract tumors: cervix, ovary	
Respiratory tract tumors: larynx, lung	
Mesenchymal tumors: fibrosarcoma, leiomyosarcoma, liposarcoma, and neurofibroma	
Renal tumors: Wilm's tumor, renal cell carcinoma	

**Table 2:** Workup for hypoglycemia.

Test	Value	Normal range
Am cortisol	28	4.30–22.40 ug/dL
TSH	1.35	0.35–3.7 uIU/mL
Thyroxine	11.8	4.5–12.1 ug/dL
Insulin	<2	<17 uIU/mL
IGF I	31	41–279 ng/mL
IGF II	326	288–736 ng/mL
